# Symptom recognition of disease and insect damage based on Mask R-CNN, wavelet transform, and F-RNet

**DOI:** 10.3389/fpls.2022.922797

**Published:** 2022-07-22

**Authors:** He Li, Hongtao Shi, Anghong Du, Yilin Mao, Kai Fan, Yu Wang, Yaozong Shen, Shuangshuang Wang, Xiuxiu Xu, Lili Tian, Hui Wang, Zhaotang Ding

**Affiliations:** ^1^Tea Research Institute, Qingdao Agricultural University, Qingdao, China; ^2^School of Science and Information Science, Qingdao Agricultural University, Qingdao, China; ^3^Tea Research Institute, Shandong Academy of Agricultural Sciences, Jinan, China; ^4^Tea Research Institute, Rizhao Academy of Agricultural Sciences, Rizhao, China

**Keywords:** tea plant, disease and pest stress, Mask R-CNN, wavelet transform, F-RNet

## Abstract

Brown blight, target spot, and tea coal diseases are three major leaf diseases of tea plants, and *Apolygus lucorum* is a major pest in tea plantations. The traditional symptom recognition of tea leaf diseases and insect pests is mainly through manual identification, which has some problems, such as low accuracy, low efficiency, strong subjectivity, and so on. Therefore, it is very necessary to find a method that could effectively identify tea plants diseases and pests. In this study, we proposed a recognition framework of tea leaf disease and insect pest symptoms based on Mask R-CNN, wavelet transform and F-RNet. First, Mask R-CNN model was used to segment disease spots and insect spots from tea leaves. Second, the two-dimensional discrete wavelet transform was used to enhance the features of the disease spots and insect spots images, so as to obtain the images with four frequencies. Finally, the images of four frequencies were simultaneously input into the four-channeled residual network (F-RNet) to identify symptoms of tea leaf diseases and insect pests. The results showed that Mask R-CNN model could detect 98.7% of DSIS, which ensure that almost disease spots and insect spots can be extracted from leaves. The accuracy of F-RNet model is 88%, which is higher than that of the other models (like SVM, AlexNet, VGG16 and ResNet18). Therefore, this experimental framework can accurately segment and identify diseases and insect spots of tea leaves, which not only of great significance for the accurate identification of tea plant diseases and insect pests, but also of great value for further using artificial intelligence to carry out the comprehensive control of tea plant diseases and insect pests.

## Introduction

Tea plant (*Camellia sinensis* (L.) *O. Kuntze*) is an important cash crop, which is widely planted in tropical and subtropical areas. In the process of tea cultivation, it is often damaged by various diseases and insect pests, resulting in heavy losses to tea production (Roy et al., 2019). Among these tea pests and diseases, brown blight, target spot, and tea coal disease often occur in tea plantations around the world and have become three important diseases causing tea production reduction. *Apolygus lucorum* is an important pest in tea plantations, which seriously affects the quality and yield of tea. The symptom judgment and analysis of diseases and pests are important for the prevention and control of diseases and pests in tea plantations.

The traditional identification of disease and pest symptoms is mainly done manually, which has some problems, such as low accuracy, low efficiency, difficult identification, and so on. For example, brown blight and target spots appeared mainly in the matured leaves and old leaves, and the symptoms were similar. Brown blight has irregular ring lines, while target spots often show obvious concentric ring lines. Moreover, in most cases, these two diseases occur at the same time, often on one leaf, which increases the difficulty of manual identification (Chen et al., 2015, 2018). Tea coal disease is caused by *Ascomycete fungi*. In the process of disease, *Bemisia spinosa* provides nutrients for the tea coal pathogen. The severity of tea coal disease is closely related to the number of *Bemisia spinosa*. In the early stage of tea coal disease, black circles appear on the front of leaves and gradually expand in the later stage. When manually discovered, it is often too late. *Apolygus lucorum* is an important pest in tea plantations. It uses the mouthparts of adults and nymphs to bite young buds, young leaves, flower buds, and young fruits. After young leaves are injured, reddish-brown or scattered black spots will appear first. With the growth of tea plants, spots become irregular holes. It mainly harms the tea plants in the morning. After biting the leaf, it transfers rapidly, and it is difficult to find. Accurate identification of the symptoms of *Apolygus lucorum* harming leaves is conducive to the early detection of *Apolygus lucorum*. Therefore, how to quickly and accurately find the symptoms of the above diseases and insect pests harming leaves is of great significance to take effective prevention and control measures.

With the rise of computer vision, high-throughput phenotypic technology has been used more in crop disease and pest identification (Jangra et al., 2021). The traditional method was usually applied to extract the color and texture, shape features of disease spots in leaves, and then distinguish the disease spots from normal leaves by adjusting the threshold (Tao et al., 2014). Researchers improved the traditional method and proposed a method based on singular value decomposition (SVD). This method extracted the comprehensive features of different color spaces, color indexes, and color to gray conversion of images and used the region growth method to segment the disease spots (Jothiaruna et al., 2019). Bao et al. (2022) proposed a classification model using UNIREP as a feature and the LIGHTGBM algorithm as a classification model. The model can effectively classify virus particle proteins, and the accuracy of the model is better than the traditional machine learning algorithm. Yang et al. (2021) proposed a novel disease-related compound identification model based on the capsule network (CapsNet). The CapsNet medal was used to identify the pneumonia-related compounds in Qingre Jiedu injection. In recent years, with the development of deep learning, feature extraction and recognition classification have brought good progress. In particular, convolutional neural network (CNN) has been widely used in feature extraction and recognition classification by researchers and has brought good progress in disease identification of wheat, rice, potato, and other crops (Ap et al., 2019; Abdu et al., 2020; Zj et al., 2021). However, most of the disease identification of these crops is to distinguish between normal leaves and diseased leaves or to identify diseases with large differences in external characteristics. It is difficult to distinguish diseases with small differences in external characteristics (Prajna, 2021). Therefore, it is difficult for CNN to directly distinguish diseases with small differences in characteristics, such as brown blight disease and target spot disease. To solve the above problems, we segmented, extracted, enhanced, recognized, and classified the images of tea plant diseases and pests.

Image segmentation is a key step from image processing to image analysis, which is widely used in plant recognition. Zhao et al. (2014) proposed a tomato leaf segmentation method based on threshold, which used the Otsu algorithm to segment the image into two parts, namely, target and background, to eliminate the interference caused by non-leaf data in the image. However, for some small disease spots, the Otsu algorithm was difficult to segment them. Therefore, the researchers proposed a cassava necrosis segmentation method based on the U-Net algorithm. The U-Net algorithm could not only segment small disease spots but also be fully trained in small sample datasets to improve data utilization efficiency (Tusubira et al., 2020). However, the structure of the U-Net network is relatively simple, and the effect of disease spots segmentation with complex features and different types is poor. Yu et al. (2021) proposed a method to segment mariculture cages from remote sensing images using Mask R-CNN. The results show that compared with U-Net, Mask R-CNN can significantly improve the segmentation accuracy and robustness of the model. Therefore, we developed a disease spots and insect spots segmentation model of tea leaves based on Mask R-CNN, which has a more complex structure and can segment more complex disease spots. At present, the Mask R-CNN algorithm has brought good progress in the fields of coffee bean leaf disease segmentation and weed species segmentation (Yang et al., 2020; Tassis et al., 2021), which has important reference value for us to engage in this research.

Image enhancement technology is an effective means to solve the problems of image blur and small differences in different object features. At present, it has become an indispensable technology in the field of computer vision. For example, Kumar and Domnic (2018) proposed a statistically based image enhancement technology, which could not only better extract leaves but also accurately calculate the number of leaves. Bao et al. (2022) proposed a novel two-fold ensemble based on method ensemble and data ensemble, which improved the accuracy of modification residue identification. Moreover, the researchers proposed an image enhancement framework, which could expand the number of training samples and provide data for target detection, semantic segmentation, image de-drying, and recognition and classification (Nesteruk et al., 2021). However, there are few studies on image feature enhancement. Therefore, we proposed an image enhancement algorithm based on the wavelet transform. The algorithm used a series of wavelets with different scales to decompose the image, so as to obtain the low-frequency and high-frequency images of the original image at the different wavelet scales. The wavelet transform is called the “image microscope” in the field of image processing because of its multi-resolution decomposition ability to decompose and strip images of information layer by layer. Therefore, the tea leaf images under the stress of diseases and insect pests can be enhanced by wavelet transform to extract more comprehensive features.

In the field of image recognition and classification, the methods of machine learning (ML) and deep learning (DL) have brought good progress, especially in improving the speed, accuracy, reliability, and scalability of disease phenotypes to accomplish diverse programmatic goals (Singh et al., 2018). Both ML and DL can be seamlessly integrated into data acquisition, data preprocessing, and data analytics for real-time high-throughput plant phenotyping (HTP) of plant traits in the field (Singh et al., 2021). For example, researchers proposed a multidimensional machine learning method. The digital image of the strawberry was converted to an ordered scale. Then, using human recognizable shape categories, the quantitative features most suitable for genetic anatomy and analysis were extracted from a variety of morphological analyses (Feldmann et al., 2020). The researchers also improved the traditional BP neural network, automatically adjusted the learning rate according to the output loss, and established a soybean disease detection model (Jiang et al., 2019). In recent years, researchers have used deep convolution neural networks (DCNN) to identify cucumber leaf diseases and compared the detection results of random forest (RF), support vector machine (SVM), and AlexNet with the detection results of DCNN. The results show that the accuracy of the DCNN model was 93.4%, which was significantly higher than other conventional classifiers (Ma et al., 2018). Researchers used multilayer convolution neural network (MCNN) to classify mango leaf diseases and found that the MCNN model had higher classification accuracy compared to other advanced methods (Singh et al., 2019). Facts show that compared with the machine learning methods, deep learning methods have better effects in the fields of image recognition and classification and have expanded to almost all the areas of plant phenotyping (Arya et al., 2022). This is mainly because deep learning can more easily perform high-precision data analysis on a large number of images (Gill et al., 2022). Among the deep learning methods, the residual network is one of the most widely used networks. The network can better fit the classification function, resulting in higher classification accuracy. Therefore, we built a four-channeled residual network (F-RNet) to classify the image of tea leaves under disease and insect stress. The network takes ResNet18 as the framework, inputs the image processed by wavelet transform into the first layer network, transforms it into a four-channeled network to extract image features, and the parameters of the network are automatically adjusted by 10-fold cross verification.

**Figure 1:**
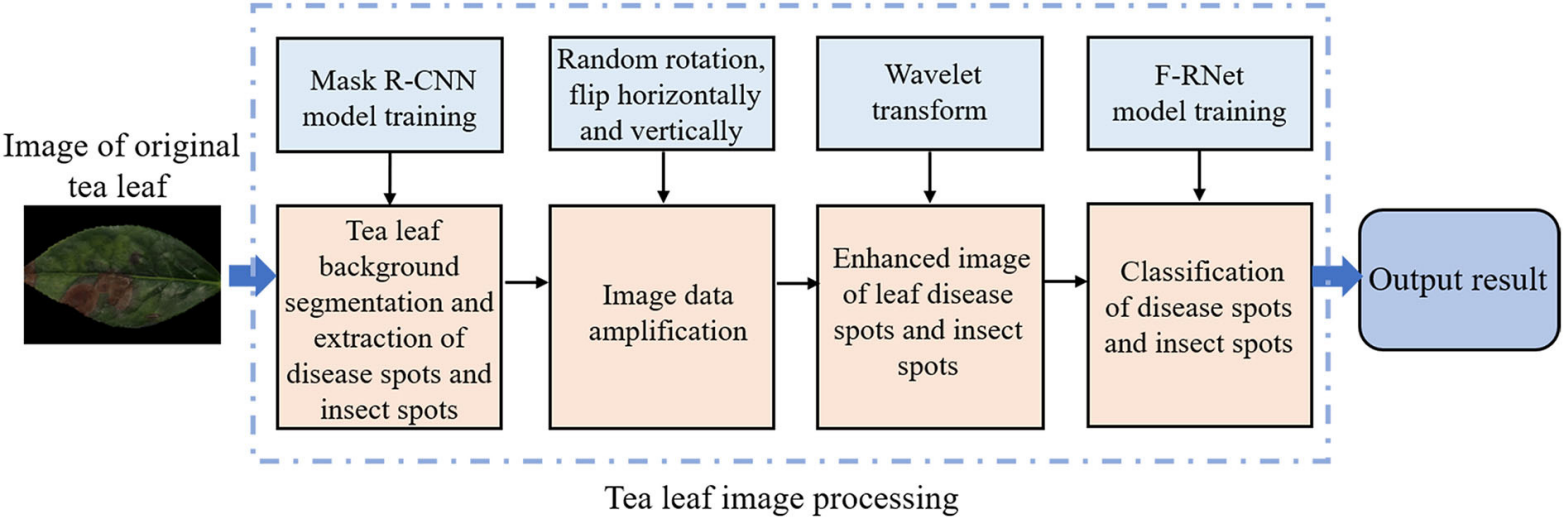
The overall framework of the model in this study.

As shown in Figure 1, it is the overall framework of the model in this study. In our implementation, the main contributions of this study are as follows:

1) A disease spots and insect spots segmentation model of tea leaves based on Mask R-CNN is proposed. The region of harmful symptoms is segmented from the image of tea leaf under disease and insect stress, and other backgrounds are removed, so as to obtain more useful information. To increase the data set, the segmented image is rotated and flipped horizontally and vertically.
2) An image enhancement algorithm based on the wavelet transform is proposed. The segmented image is transformed by wavelet transform, and the image is gradually multi-scale refined by scaling and translation operations to obtain a low-frequency image and three high-frequency images, so as to extract more comprehensive disease spots and insect spots information.
3) A detection model of a four-channeled residual network (F-RNet) is proposed. The low-frequency and high-frequency images processed by wavelet transform are input into a four-channeled convolutional neural network to extract image features. At the same time, the imagefolder function is overloaded, and the images are selected as the training set and test set by means of 10-fold cross-verification to train the network and adjust the parameters.

## Materials and Methods

### Data acquisition

**Figure 2:**
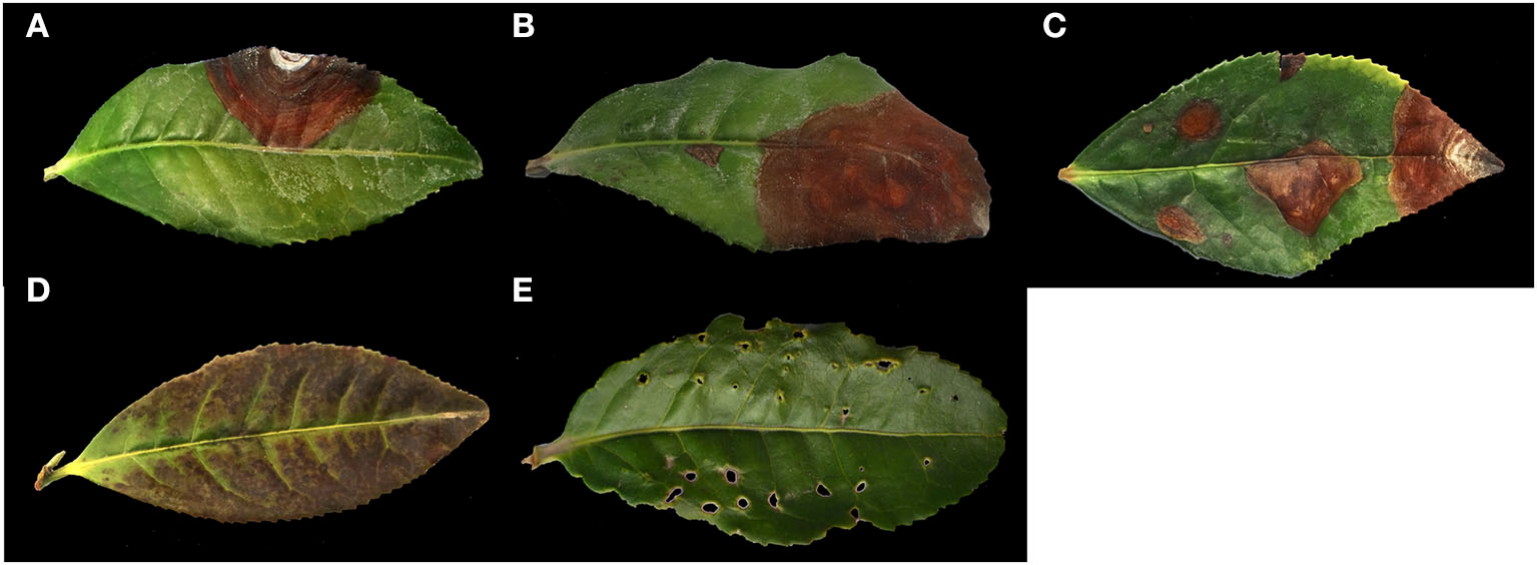
The collection of the original photographs. **(A)** BB; **(B)** TS; **(C)** BB and TS; **(D)** TC; **(E)** AL.

In April 2021, the images of tea leaves under disease and insect stress were collected at Rizhao Tea Research Institute, Shandong Province, China (35°40′N, 119°33E). The varieties of tea are Longjing 43, Jinxuan, and Zhongcha 108. The images were taken by a Canon EOS 6D digital camera under natural light conditions, and the shooting angle and shooting distance were random. The images were saved in JPG format with a resolution of 6,000 × 4,000. In this study, the following **four** kinds of images of tea leaves under disease and insect stress with a high incidence rate were selected: brown blight (BB), target spot (TS), tea coal disease (TC), and *Apolygus lucorum* endangers leaves (AL). Among them, there is less difference in phenotypic characteristics between BB and TS, and these two diseases often exist in tea leaves at the same time. We collected about 1,200 images in total, and five types of images are shown in Figure 2.

### Data labeling

**Figure 3:**
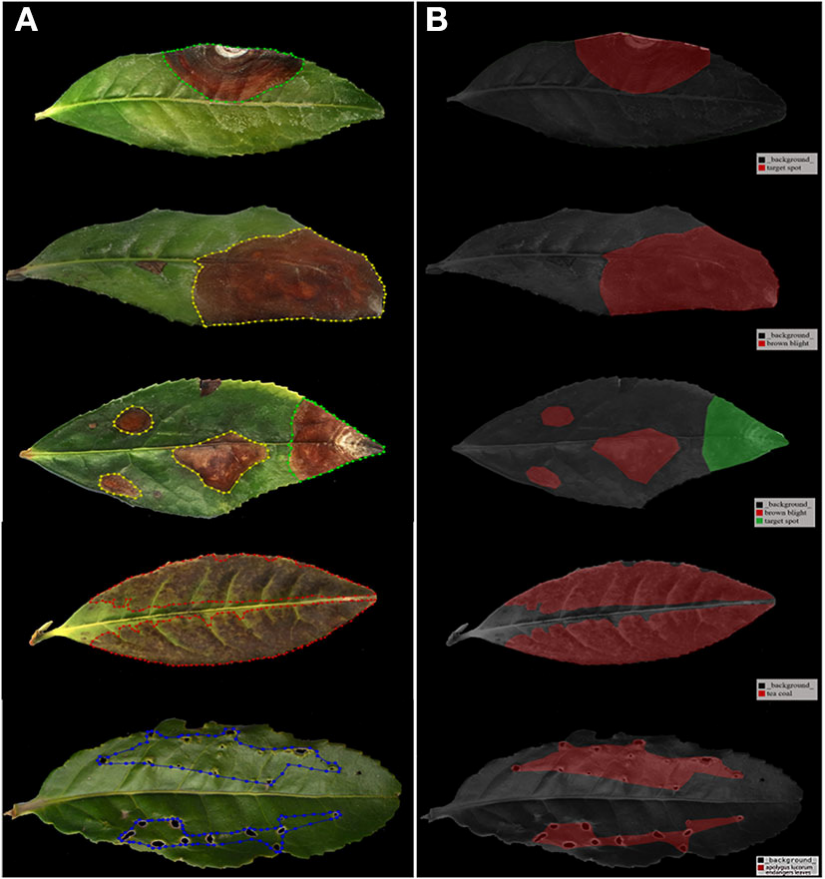
Labeled images sample. **(A)** The images in the process of leaves labeling and **(B)** images after leaves marking.

The training sets used in the Mask R-CNN network must be labeled. Therefore, the Labelme software was used to construct training sets to manually mark the images of tea leaves under disease and insect stress. The Labelme software was developed by the MIT Computer Science and artificial intelligence laboratory. First, the symptom areas of disease and pest damage were marked with different colors, and each symptom area was classified with different color labels. Among them, yellow, green, red, and blue color labels represent BB, TS, TC, and AL, respectively. Areas beyond the mark were treated as background. Then, the labeling data was saved in a JSON file corresponding to the original photo. Figure 3A shows the images in the process of leaf labeling. We converted the JSON format marking data into visual images to obtain the leaf marking image in Figure 3B. Here, you could see that the different target parts were covered with different color masks, and the labels we marked were displayed in the lower right corner of the image. Next, we converted the JSON format of the tag data into the COCO dataset format and input it into the neural network for training.

### Mask R-CNN

**Figure 4:**
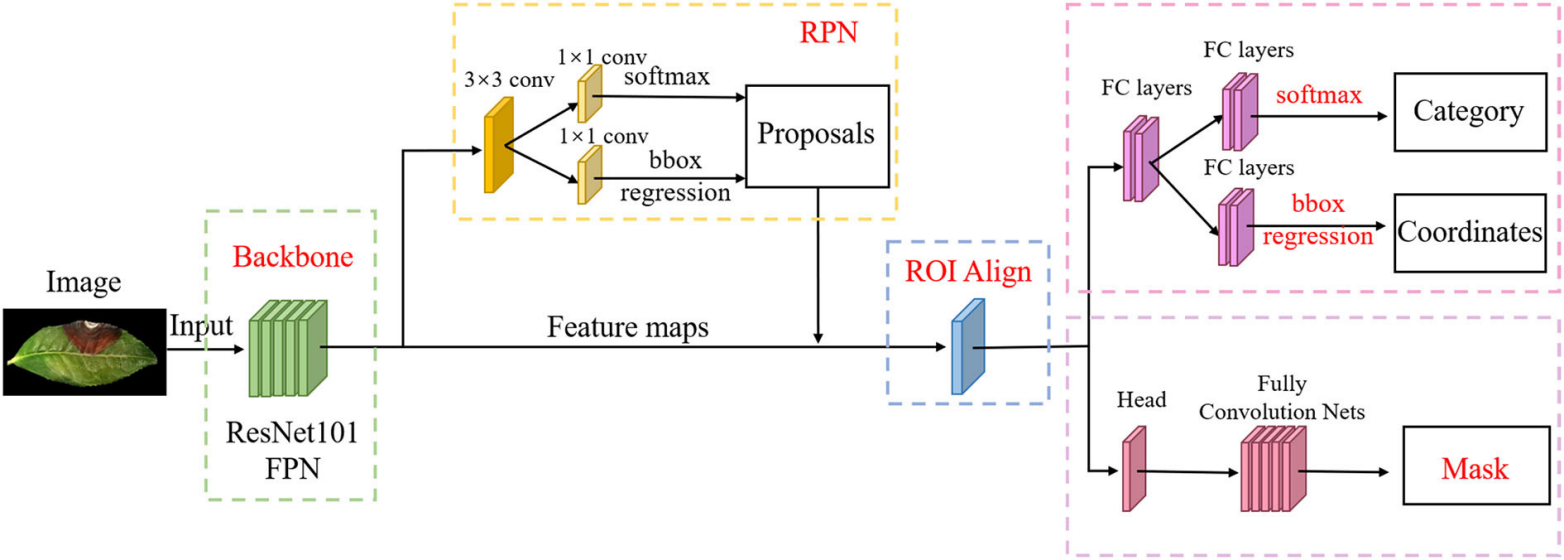
Structure of Mask R-CNN.

To further classify the disease spots and insect spots on tea leaves, we extracted the disease spots and insect spots from tea leaves by using the Mask R-CNN network. The Mask R-CNN network could be divided into five main structures (He et al., 2017). We drew the Mask R-CNN network structure as shown in Figure 4. The five structures in Mask R-CNN are explained as follows:

(1) Backbone framework: In this study, ResNet101 and feature pyramid network (FPN) are used as the backbone framework to extract the image features of tea leaf disease spots and insect spots. Resnet101 is a mainstream convolutional neural network, which can extract features from images. We used ResNet101 to convolute the disease spots and insect spots images of tea leaves 101 times and extracted the low-level and high-level features of the disease spots and insect spots images. Then, FPN is used to fuse the feature images from the bottom to the top, so as to achieve the best extraction effect. FPN can improve the accuracy and speed and generate higher quality feature map pyramids.
(2) Region proposal network (RPN): Using a sliding window, the RPN can receive the feature map extracted from the backbone structure, and the images are divided into two categories, namely, target disease and insect spots and background. Then, the disease spots and insect spots are selected by the boxes that fit the size of disease spots and insect spots as far as possible. If the predicted boxes overlap too much in a region, the RPN prediction will retain the box with the highest foreground score and discard the remaining boxes. At this time, the target disease spots and insect spots areas and the background can be roughly distinguished, and it is impossible to carry out detailed classification and segmentation of the target disease spots and insect spots.
(3) Region of interest (ROI) align: ROI align is used to receive the region of interest from the RPN. Using the bilinear interpolation, the feature map of the region of interest is cut by pooling and sent to two branches. One branch network is a region of interest classifier and a border regression, and the other branch network is a mask generation network composed of the full convolutional networks (FCN).
(4) Box regression and classification: ROI classifier and border regression are used for target disease spots and insect spots recognition, and both of them are composed of a full connection layer. The ROI classifier classifies the ROI into specific disease spots and insect spots. The border regression adjusts the center point position and aspect ratio of the region of interest to detect disease spots and insect spots more accurately.
(5) Segmentation mask: The mask-generating network is composed of fully connected network (FCN). The network can generate a mask consistent with the size and shape of the target disease spots and insect spots, and segment the images of the target disease spots and insect spots. Finally, the mask images are combined with the recognition result to obtain an image containing the target disease and insect spot category and segmentation mask.

The loss function of Mask R-CNN is as follows:


(1) $$\begin{array}{r} L\;=L_{cls}\;+\;L_{box}\;+\;L_{mask} \end{array}$$

***L***_***cls***_ and ***L***_***box***_ are consistent with the classification and regression losses defined in Faster R-CNN (Ren et al., 2017). The mask branch has ***k* × *m***^**2**^ dimensions of output for each ROI. Represents *k* binary masks with resolution ***m* × *m***. ***L***_***mask***_ is the average binary cross loss. For an ROI belonging to the k category, ***L***_***mask***_ considers only the k mask.

### Two-dimensional discrete wavelet transforms of disease and insect damage spots image

**Figure 5:**
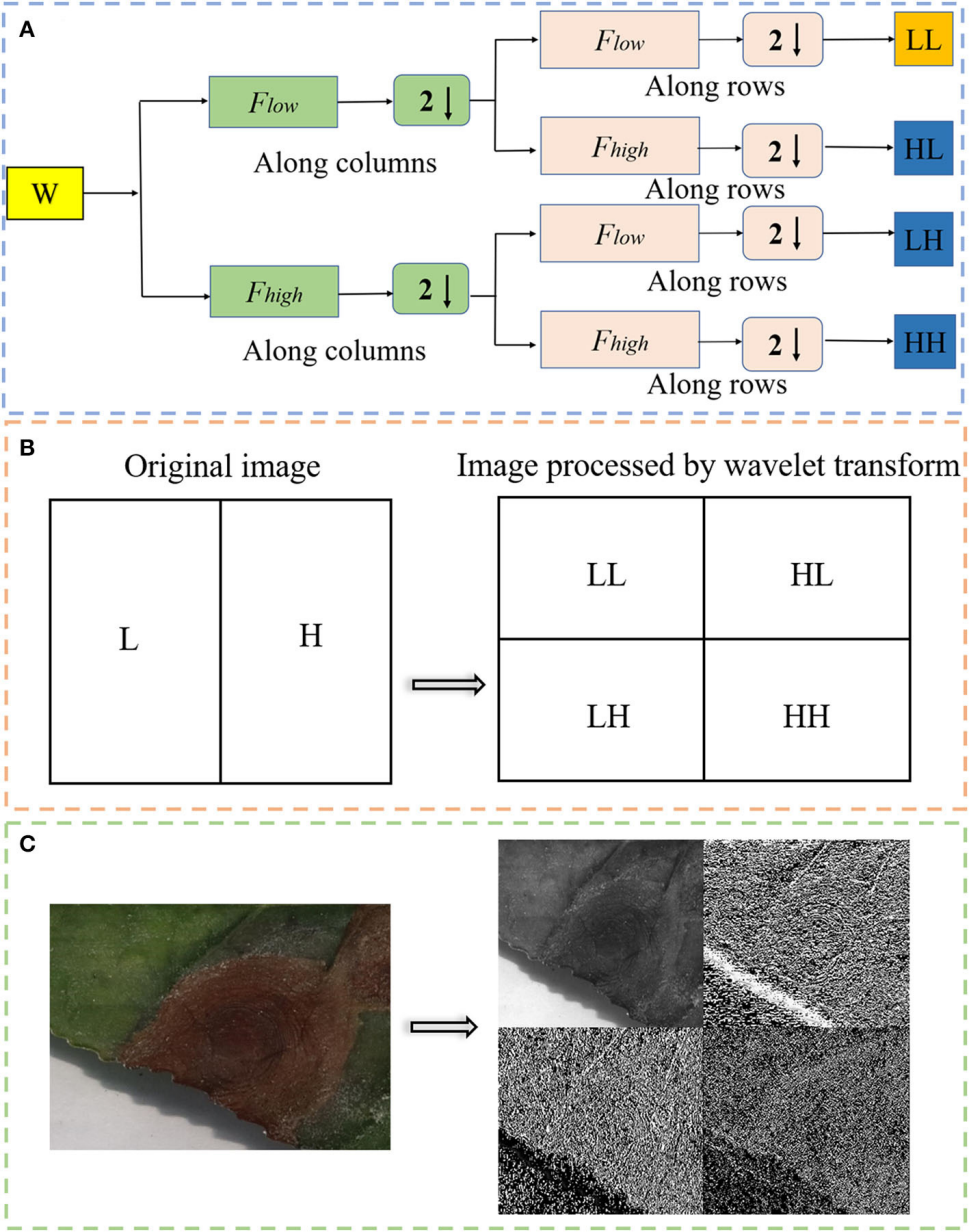
**(A)** The image signal is separated by wavelet transform according to low frequency and high frequency. “W” is the original image; “***F***_***high***_” is the high frequency component; “***F***_***low***_” is the low frequency component; **(B)** schematic diagram of wavelet transform; and **(C)** real image of wavelet transform.

To better extract the characteristic information of tea leaf disease and insect damage spots, the images were enhanced by wavelet transform. The wavelet transform first converts the image into a signal, and then transforms the signal by selecting the appropriate wavelet base and wavelet decomposition scale in order to obtain the low-frequency coefficients and high-frequency coefficients. Finally, the signal is separated according to the low-frequency coefficient and high-frequency coefficient to obtain four components, as shown in Figure 5. The four components are described as follows:

(1) LL component is a wavelet coefficient generated by convolution of low-pass filter in row direction and column direction. It is an approximate representation of image.
(2) HL component is a wavelet coefficient generated by the convolution of a low-pass filter in row direction and then the convolution of a high-pass filter in the column direction. It represents the characteristics of the horizontal direction of the image.
(3) LH component is the wavelet coefficient generated by the convolution of a high-pass filter in the row direction and then the convolution of a low-pass wavelet filter in the column direction. It represents the characteristics of the vertical direction of the image.
(4) HH component is the wavelet coefficient generated by convolution of high pass filter in the row and column directions. It represents the characteristics of the diagonal edge of the image.

A discrete wavelet transform is a mathematical method for time-frequency analysis of discrete-time signals. Its main idea is the multi-resolution analysis process. In the process of two-dimensional image decomposition by discrete wavelet transform, LL component can cycle many times until it meets the requirements. In this study, the LL component only circulates once. For the image *f* (x, y) with a size of M × N, the formula of discrete wavelet transform is as follows:


(2) $$W\varphi \left(j_{0},m,n\right)=\;\frac{1}{\sqrt{MN}}\sum_{x=0}^{M-1}\sum_{y\;=\;0}^{N-1}f\left(x,y\right)\varphi_{j^{0},m,n}(x,y)$$

***j*_0_** is an arbitrary starting scale; Wφ is the approximate coefficient in the scale ***j*_0_** ; $\varphi_{j^{0},m,n}(x,y)$ represents a scale function.

### F-RNet

**Figure 6:**
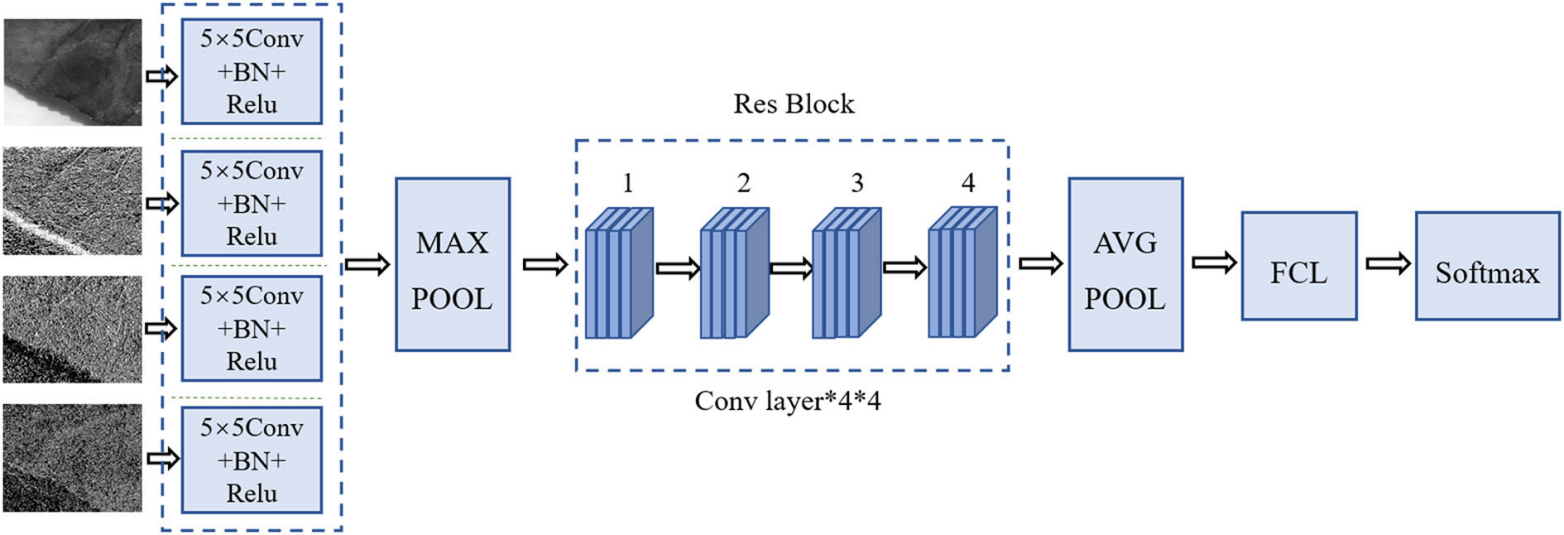
Structure of F-RNet.

In this study, we proposed a novel deep learning model called F-RNet to classify disease spots and insect spots images. Compared with the traditional convolutional neural network, the network adds residual blocks, which can solve the problems of gradient disappearance and difficult network training in the depth network. When the network layer is too deep, the residual blocks are connected by the identity map that introduces *A*, and the network degradation is solved by fitting the *X*(*A*) in *Y*(*A*) = *X*(*A*)+*X* to 0. Owing to the introduction of *A*, the derivative value is invariably >1 in the back propagation process, which prevents the gradient of the neural network from disappearing. F-RNet is based on ResNet18, as shown in Figure 6. The BN is the batch regularization processing, the Relu is the activation function, the MAX POOL represents the maximum pool operation, AVG POOL represents the global average pool layer operation, and Rebslock-1 to Rebslock-4 represent residual blocks.

To enhance the underlying features and enable the network to achieve better classification effects, in the first layer network, four-channeled convolution layers are used to extract the characteristics of disease spots and insect spots. The input data size of the ResNet18 neural network is 224 × 224 × 3. Therefore, it is necessary to preprocess images of different frequencies before inputting data to ensure smooth input. The images of four frequencies processed based on the wavelet transform are input to four 5 × 5 filters for convolution. After feature fusion, nonlinear transformation, and maximization, the images are input to Resblock-1, and each residual block contains four convolution layers. After the continuous convolution of residual blocks, the number of channels of the image pixel matrix becomes deeper and deeper. Finally, the size of the image pixel matrix is input to the full connection layer FC, and the corresponding category probability is output by the Softmax classifier.

To simplify the tedious operation of making data sets, we combined the image folder function in the Pytorch framework with the 10-fold cross-validation in statistics, overloaded the image folder function, made the obtained pictures into 9 training sets and 1 test set in the way of 10-fold cross-validation, packaged them into the DATA dictionary in the way of K and Key values, replacing the traditional iterative training method, and further improved the training efficiency of the network. For each iteration cycle, we will get the accuracy and loss rate of the results for 10 times, take the average value as the estimation of the model accuracy, and then automatically discard the parameters with low accuracy and retain the parameters with high accuracy.

### Overall framework

**Figure 7:**
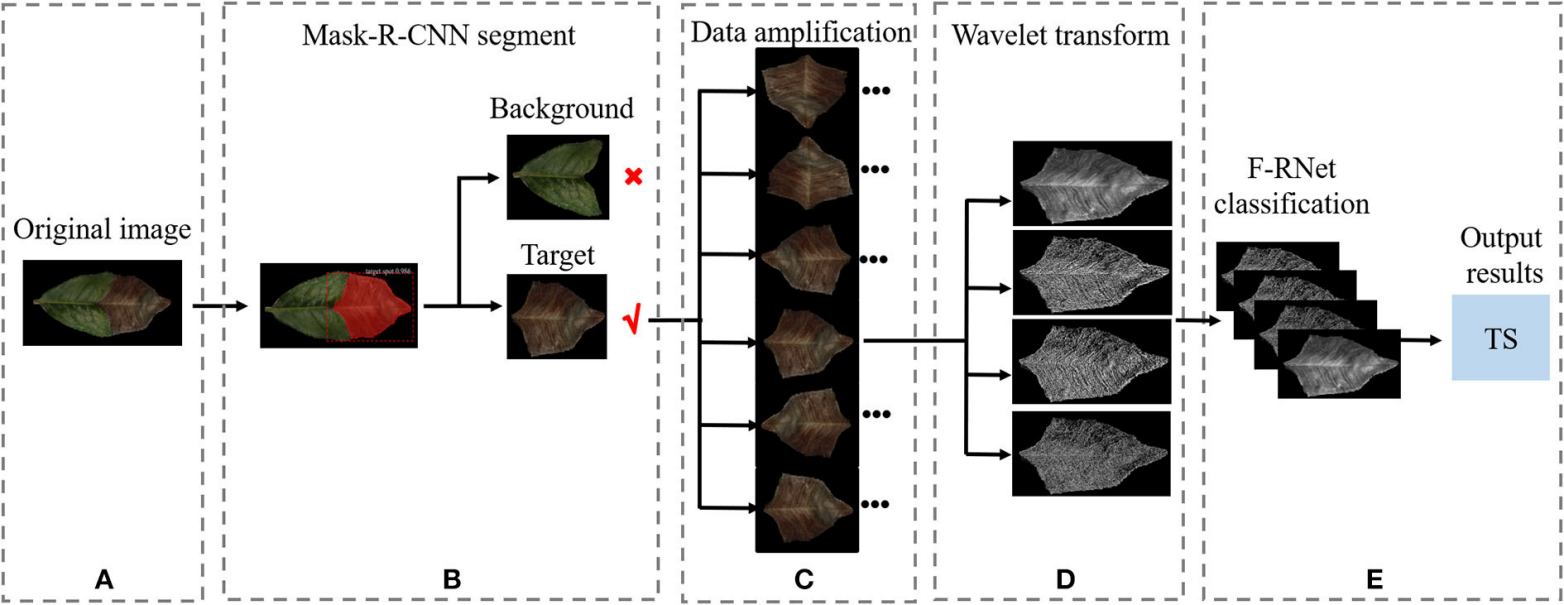
A legend to summarize the overall framework of this study. **(A)** Original image; **(B)** Mask R-CNN segment; **(C)** Amplification; **(D)** Wavelet transform; **(E)** F-RNet classification.

To more clearly express the overall idea of this study, we added a legend to summarize the overall framework of this study (Figure 7). Figure 7A shows the original image of tea leaf target spot disease, and Figure 7B shows the target and background segmented by the Mask R-CNN model. The background image is discarded, and the target image is rotated and flipped horizontally and vertically to form four images (Figure 7C). The amplified image is processed by wavelet transform, and four images with different frequencies are obtained (Figure 7D). These four images with different frequencies are simultaneous input to F-RNet for classification (Figure 7E). Finally, the classification result is TS.

## Results and analysis

### Experimental environment and evaluation method

The data processing environment of this experiment are as follows:

Hardware: Processor: Inter Xeon CPU E5-2640 V4 @ 2.4GHZ 2.40GHZ (two processors); RAM: 128 GB; Software environment: CUDA Toolkit 10.1; CUDN V7.6.0; Python 3.8; Pytorch-GPU 1.6.0; Operating system: Windows 10.

To evaluate the overall probability of the model correctly classifying all disease spots and insect spots, accuracy index was adopted. To evaluate the effectiveness of the model in single disease spots or insect spots detection, precision and recall indices were adopted. To evaluate the comprehensive performance of disease spots and insect spots detection model, F1-score index was adopted. The formula is as follows:


(3) $$\begin{array}{rcl} \text{Accuracy} & = & \frac{\text{TP + TN}}{\text{TP + FP + TN + FN}} \end{array}$$

(4) $$\begin{array}{rcl} \text{Precision} & = & \frac{\text{TP}}{\text{TP + FP}} \end{array}$$

(5) $$\begin{array}{rcl} \text{Recall} & = & \frac{\text{TP}}{\text{TP + FN}} \end{array}$$

(6) $$\begin{array}{rcl} \text{F1 - score} & = & 2\times \frac{\text{precision}\times \text{recall}}{\text{precision + recall}} \end{array}$$

“*TP*” is the number of positive samples correctly classified and located. “*TN*” is the number of negative samples correctly classified and located. “*FP*” is the number of negative samples wrongly classified as positive samples. “*FN*” is the number of positive samples wrongly classified as negative samples (Xie et al., 2018).

### Mask R-CNN model segmentation of disease spots and insect damage spots on tea leaves

#### Training of Mask R-CNN model

**Figure 8:**
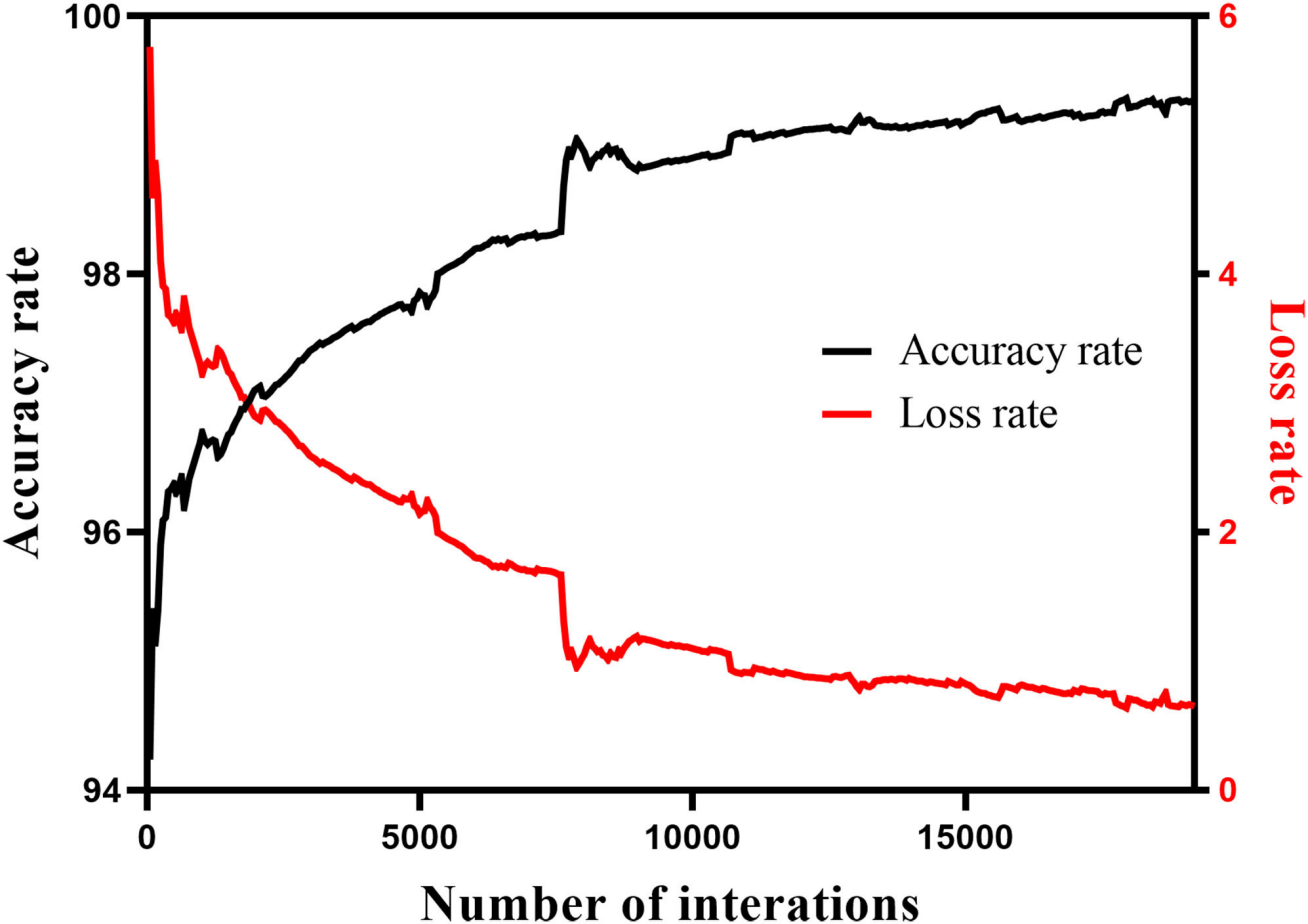
Variation trend of loss rate and accuracy rate in the training process of Mask R-CNN model.

In this study, the 1,200 images were divided into training sets and test sets according to the method of five-fold cross-validation. The learning rate was 0.001, the batch size was 1, the epoch was 20, and the momentum was 0.9. Figure 8 shows the change trend of the loss rate and accuracy rate in the training of the Mask R-CNN model.

#### Mask R-CNN model extraction of overall disease spots and insect spots

In this study, we first analyzed the two categories of disease spots and insect damage spots (DSIS) and non-disease spots and insect damage spots (NDSIS). Table 1 shows the detection results of Mask R-CNN on the whole DSIS area. We can see that the precision rate of the model is 94.8%, the recall rate is 98.7%, and the F1 score is 96.7%. This shows that the Mask R-CNN model can well distinguish DSIS and NDSIS, and almost DSIS can be identified. This provides a basis for further research on the classification of DSIS.

**Table 1:** Mask R-CNN test results for the whole area of disease spots and insect spots.

**Type**	**Model**	**Precision**	**Recall**	**F1-score**
DSIS	Mask R-CNN	94.8%	98.7%	96.7%

#### Mask R-CNN model classification of disease spots and insect spots

**Figure 9:**
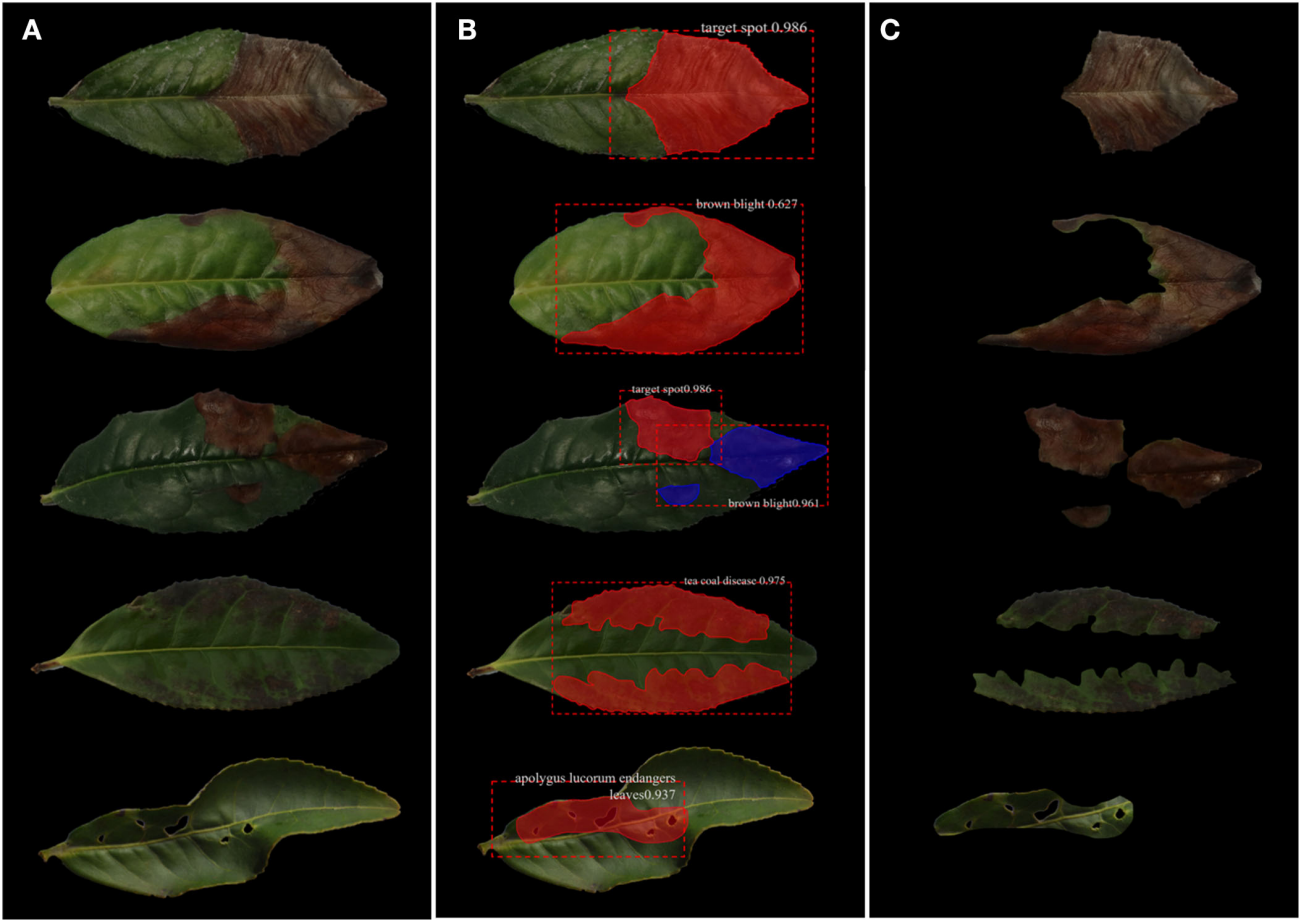
Segmentation process of tea leaf disease spots and insect damage spots. **(A)** Original image; **(B)** identified image; and **(C)** segmented image.

To explore whether the Mask R-CNN model can well classify disease spots and insect spots, we compared and analyzed the recognition results of disease spots and insect damage spots, as shown in Table 2. The results show that the model has achieved good results in the identification of TC and AL. The F1-score of TC and AL identified by the Mask R-CNN model is 88.3 and 95.3%, respectively, indicating that this model can well distinguish TC and AL. However, the F1 scores of BB and TS are 61.1 and 66.6% respectively, and the recognition accuracy is lower than 60%, indicating that the Mask R-CNN model cannot effectively distinguish the two disease spots. This may be because the textures of the two disease spots are too similar. It is easy to confuse the features of these two disease spots when extracting the features of the two disease spots. The final results show that Mask R-CNN cannot distinguish these four DSIS well. Therefore, we proposed a new F-RNet network to better classify disease spots and insect spots. Figure 9 shows the segmentation process of tea leaves with disease spots and insect spots.

**Table 2:** Mask R-CNN model recognition results of disease spots and insect spots.

**Types of disease spots and insect spots**	**Precision (%)**	**Recall (%)**	**F1-score(%)**
BB	50.1	78.3	61.1
TS	55.9	81.4	66.6
TC	89.4	87.2	88.3
AL	92.3	98.5	95.3

### F-RNet model classification of disease spots and insect damage spots

#### Training of F-RNet model

**Figure 10:**
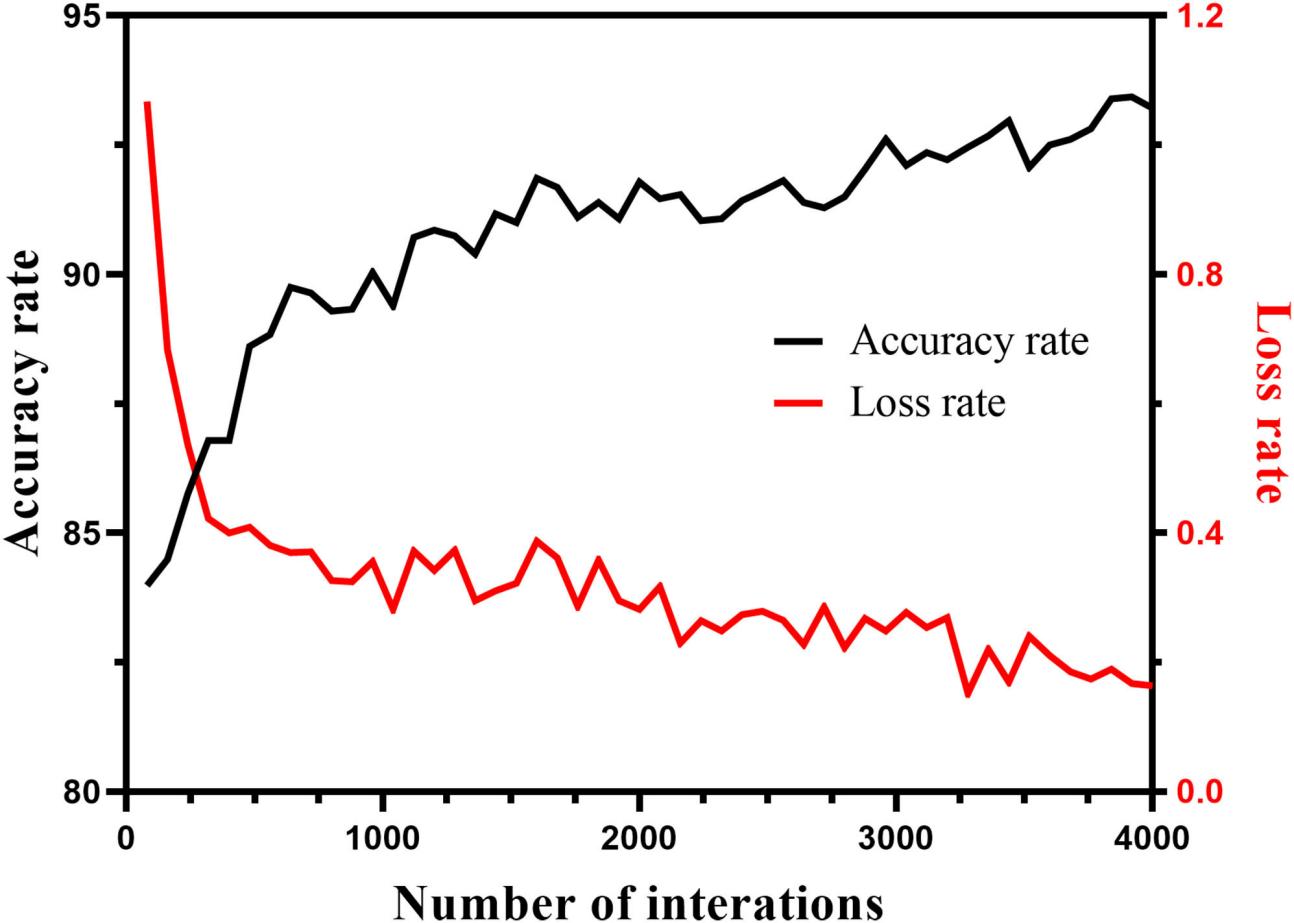
Variation trend of loss rate and accuracy rate in the training process of F-RNet model.

To increase the number of images, the segmented image by Mask R-CNN was subjected to data amplification, namely, rotation, horizontal flip, and vertical flip. F-RNet network used 10-fold cross-validation to select images as training sets and test sets for training and automatically adjust parameters. The initial learning rate was 0.001, the epoch was 90, the batch size was 64, and the momentum parameter was 0.9. To prevent overfitting of the model, the learning rate was reduced by one-third for every 27 iterations, and the final learning rate was 0.000037. In addition, the F-RNet model used the Adam optimizer, which had the advantages of fast convergence and easy adjustment of parameters. Figure 10 shows the change trend of the loss rate and accuracy rate in the training process of the F-RNet model.

#### F-RNet model classification of disease spots and insect spots

To improve the accuracy of the network model in the classification of disease spots and insect spots, we built a four-channeled residual network (F-RNet) based on the ResNet18 network and wavelet transform to classify disease spots and insect spots images segmented by the Mask R-CNN model in detail. To verify whether our improved model can improve the performance of the network, we tested the F-RNet network and other models (i.e., SVM, AlexNet, VGG16, and ResNet18) under the same test environment and the same test set. With the increase of network depth, the training loss decreases and the network performance becomes more optimized. Table 3 shows the test accuracy of disease spots and insect spots images in different models. Among them, the classification accuracy of the SVM model is 65%, which is the worst of the five models, which shows that the method of deep learning is significantly better than the traditional machine learning method. The classification accuracy of ResNet18 is 82%, which shows that the residual network model is better than the traditional CNN model, which is consistent with the previous research results (Hati and Singh, 2021). The classification accuracy of our proposed F-RNet model is 88%, which is the highest classification accuracy among the five models, while the classification accuracy of ResNet18 is 82%, which shows that the generalization ability of the improved network model has been improved.

**Table 3:** Test accuracy of disease spots and insect spots images in different network models.

**Model**	**Input size**	**Number of test set images**	**Accuracy (%)**
SVM	256 × 256	480	65
AlexNet	256 × 256	480	73
VGG16	256 × 256	480	80
ResNet18	256 × 256	480	82
F-RNet	256 × 256	480	88

**Figure 11:**
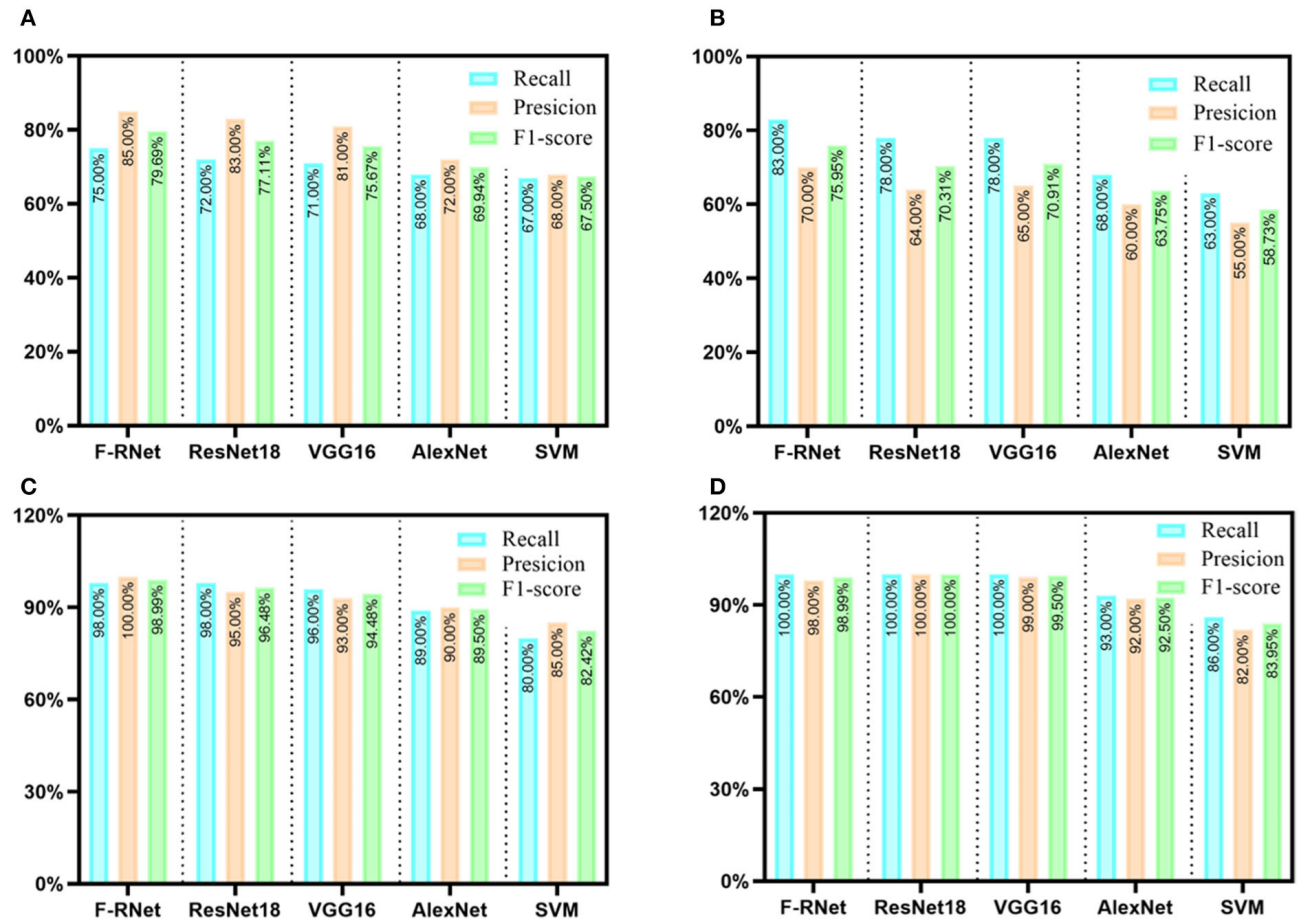
Evaluation results of different disease spots and insect spots by different network models. **(A)** BB; **(B)** TS; **(C)** TC; and **(D)** AL.

To further compare the recognition performance of different models for single disease spots and insect spots, we evaluated five models through precision, recall, and F1-score indicators, as shown in Figure 11. The results show that for BB, TS, and TC classification, the three evaluation indexes of the F-RNet model are the highest. For the classification of AL, the three evaluation indexes of the ResNet18 model are the highest. This may be related to the obvious characteristics of AL, and the performance of relatively shallow networks is better. However, the difference between the F1-score of the ResNet18 model and the F-RNet model is not large (only 3%). Therefore, the F-RNet model has the best comprehensive performance in classifying disease spots and insect spots.

## Discussion

### Mask R-CNN model can accurately extract disease spots and insect spots from tea leaves

Mask R-CNN is a target instance segmentation model proposed by He Kaiming et al. on the basis of Faster R-CNN (Girshick et al., 2015; He et al., 2017). The model adds a branch for predicting the target mask in parallel with the existing Rox identification branch of Faster R-CNN. Previously, researchers used the Mask R-CNN model to segment the leaves of different plants and then used the VGG16 network to classify the leaves, so as to realize the identification of plant varieties (Yang et al., 2020). In this study, the effect of DSIS segmentation using the Mask R-CNN model is consistent with that of plant leaves segmentation. From able 2, it can be seen that the Mask R-CNN model has poor results in identifying four DSIS areas, especially identifying BB and TS, with an accuracy of only about 50%. However, the Mask R-CNN model is effective in segmenting DSIS and NDSIS. This is very important to ensure that the whole DSIS areas can be accurately segmented, which provides basic work for further identification of the DSIS. In this study, due to the high similarity of disease spots, the classification effect of Mask R-CNN is not ideal, but the overall detection rate of disease spots and insect spots is very good. Therefore, we introduced a new classification model to make up for the deficiency of Mask R-CNN in classification.

### F-RNet model has good robustness for the classification of disease spots and insect spots

**Figure 12:**
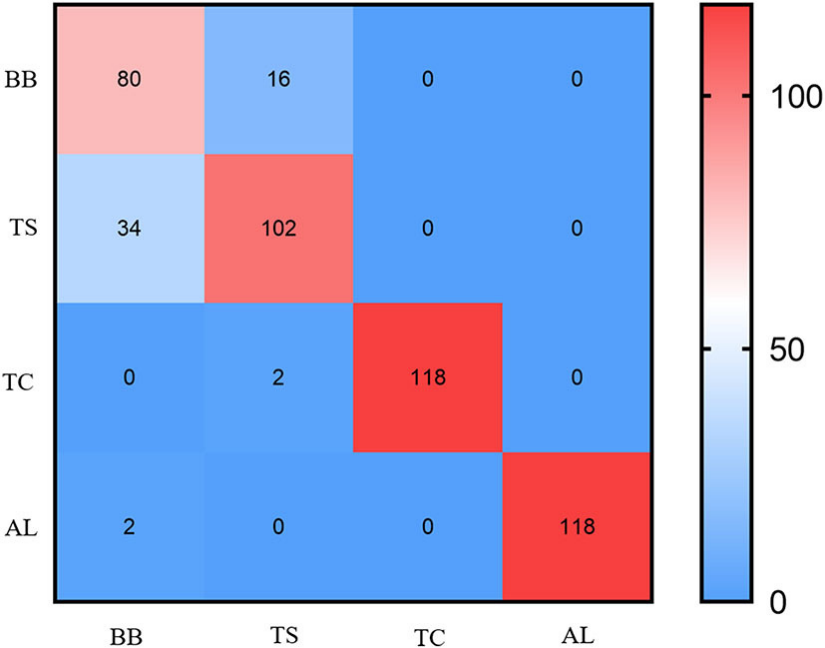
Confusion matrix of tea leaf DSIS detection model based on F-RNet.

We used the confusion matrix to observe the misclassification between DSIS, as shown in Figure 12. The results showed that about 20% of TS were incorrectly classified as BB. There may be two reasons for this situation. First, the texture features of the two disease spots are too similar, and the model is difficult to identify; second, when Mask R-CNN segments the disease spots areas, one image contains these two kinds of disease spots, which leads to the confusion of the features of these two kinds of disease spots in the extraction of disease spots features by F-RNet. Therefore, in a subsequent study, we can increase the number of TS images and collect images with more obvious texture features to further optimize the model. For the identification of BB, TC, and Al, F-RNet can be classified accurately with less misclassification. The overall accuracy of the model is 88%. This shows that the model has good robustness and can accurately classify the disease spots.

### F-RNet model can improve the accuracy of classifying disease spots and insect spots

We compared the classification accuracy of the F-RNet model with that of other models (SVM, AlexNet, VGG16, and ResNet18). The results show that the classification accuracy of the F-RNet model is greatly improved for TC and AL, and the classification accuracy of TC and AL is slightly improved. This shows that the F-RNet model has great potential to identify complex and similar disease spots. The reasons why F-RNet is superior to other models are analyzed. On the one hand, the four-channeled network is more comprehensive than the single channel network in extracting the features of disease spots. Previously, researchers built a two-channeled residual network to identify tomato leaf diseases. The results show that the classification accuracy of B-ARNet model (double channel residual network) is higher than that of the ResNet50 model (single channel residual network) (Chen et al., 2020). On the other hand, wavelet transform can reduce or remove the correlation between different features of disease and insect damage spot image and enlarge the main texture features of the disease and insect damage spots by selecting an appropriate filter. In future research, we can deeply mine and analyze the technology of wavelet transform. In this study, the wavelet transform decomposes the image only once. In the follow-up research, we can decompose the image many times to deeper study the impact of the wavelet transforms on the performance improvement of the model.

## Conclusion

In this study, a recognition framework of disease and insect damage symptoms in tea leaf images based on Mask R-CNN, wavelet transform, and F-RNet is proposed. First, we used the Mask R-CNN model to segment disease spots and insect spots from leaves. Then, the two-dimensional discrete wavelet transform is used to enhance the features of disease spots and insect spots images, so as to obtain the images with four frequencies. Finally, the images of four frequencies are simultaneously input into the four-channeled residual network (F-RNet) to identify the disease and insect damage symptoms. The results showed that the Mask R-CNN model could detect 98.7% of DSIS, which ensures that almost all disease spots and insect spots can be extracted from leaves. The accuracy of the F-RNet model is 88%, which is higher than that of other models (e.g., SVM, AlexNet, VGG16, and ResNet18). Therefore, this experimental framework can accurately segment and identify disease spots and insect spots in tea leaves, which is not only of great significance for the accurate identification of tea plant diseases and insect pests but also of great value for further using artificial intelligence to carry out the comprehensive control of tea plant diseases and insect pests.

## Data availability statement

The dataset and source code has been uploaded to GitHub: https://github.com/matterport/Mask_RCNN, https://github.com/Du553/F-RNet.

## Author contributions

HL carried out the experiment, collected and organized data, used the Mask R-CNN and F-RNet models to analyze the data, and wrote the manuscript. HS and AD built Mask R-CNN and F-RNet models. YM provided the modification of the article's picture. SW, XX, and LT identified diseases. HW collected images. ZD, YW, and KF proposed the hypothesis for this work, designed the experiment, helped organize the manuscript structure, and directed the study. YS participated in the design of the experiment and directed the study. All authors contributed to the article and approved the submitted version.

## Funding

This study was funded by the Significant Application Projects of Agriculture Technology Innovation in Shandong Province (SD2019ZZ010), the Technology System of Modern Agricultural Industry in Shandong Province (SDAIT-19-01), the Special Foundation for Distinguished Taishan Scholar of Shandong Province (No. ts201712057), the Livelihood Project of Qingdao City (19-6-1-64-nsh), and the Project of Agricultural Science and Technology Fund in Shandong Province (2019LY002, 2019YQ010, and 2019TSLH0802).

## Conflict of interest

The authors declare that the research was conducted in the absence of any commercial or financial relationships that could be construed as a potential conflict of interest.

## Publisher's note

All claims expressed in this article are solely those of the authors and do not necessarily represent those of their affiliated organizations, or those of the publisher, the editors and the reviewers. Any product that may be evaluated in this article, or claim that may be made by its manufacturer, is not guaranteed or endorsed by the publisher.
